# Navigierte und minimalinvasive Schraubenosteosynthese einer Talusfraktur

**DOI:** 10.1007/s00113-024-01513-2

**Published:** 2024-12-17

**Authors:** Dominik M. Haida, Thorsten Möhlig, Stefan Huber-Wagner

**Affiliations:** 1 Klinik für Unfallchirurgie, Technische Universität München, Klinikum rechts der Isar, Ismaninger Straße 22, 81675 München, Deutschland; 2 Klinik für Unfallchirurgie, Wirbelsäulenchirurgie und Alterstraumatologie, Diakonie-Klinikum Schwäbisch Hall, Diakoniestraße 10, 74523 Schwäbisch Hall, Deutschland

**Keywords:** Navigation, Talus, Fuß, Hybrid-OP, Unfallchirurgie, Navigation, Talus, Foot, Hybrid operating room, Trauma surgery

## Abstract

**Operationsziel:**

Das Ziel dieser Operation ist es, eine mehrfragmentäre und undislozierte Talusfraktur (Corpus und Hals) navigiert und minimalinvasiv mittels Schraubenosteosynthese gegen eine sekundäre Dislokation abzusichern.

**Indikation:**

Aufgrund des jungen Alters des Patienten im Beispielfall und auch der Gefahr einer möglichen sekundären Dislokation wurde die Entscheidung zur Operation getroffen.

**Kontraindikationen:**

Weichteilschwellung, Wundinfektion und Allergien gegen das Osteosynthesematerial.

**Operationstechnik:**

In dem Online verfügbaren Video (auf Englisch) erfolgt eine detaillierte Darstellung der einzelnen Operationsschritte.

Präoperative CT-Bildgebung und Schraubenplanung. Befestigung der Referenzeinheit. 1. CBCT-Scan, Bildfusion und Fusionskontrolle. Planung der minimalinvasiven Hautschnitte. Hautschnitt, navigierte Bohrungen und Einführen der Kirschner(K)-Drähte. 2. CBCT-Scan und Lagekontrolle der K‑Drähte, wenn nötig Feinjustierung dieser. Einbringen der Schrauben. 3. CBCT-Scan mit anschließender Lagekontrolle der Schrauben, wenn nötig, ein Nachziehen dieser.

Durchgeführt in der „Robotic Suite“ (Brainlab, München, Deutschland) unter der Verwendung folgender Elemente: Navigationseinheit „Curve Navigation System“, fahrbarer robotischer 3D-Computertomograph (CBCT), „Loop-X“ und der Wandmonitor „BUZZ“.

**Weiterbehandlung:**

Postoperatives Röntgen und CT zur Lagekontrolle der Implantate. Teilbelastung des Fußes mit 10 kg bei Sohlenkontakt für 6 Wochen. Physiotherapie mit aktiver und passiver Gelenkmobilisation. Thromboseprophylaxe mit Enoxaparin-Natrium. Metallentfernung fakultativ nach ca. einem Jahr.

**Evidenz:**

Navigierte Operationen sind Routine, bisher v. a. im Bereich der Wirbelsäule. Dieser Beitrag zeigt, dass navigierte Eingriffe an den Extremitäten erfolgreich in Hybrid-OP durchgeführt werden können.

**Video online:**

Die Online-Version dieses Beitrags (10.1007/s00113-024-01513-2) enthält das anschauliche Video zur Operationstechnik.

## Abstract (en)

**Objective of surgery:**

The aim of this surgery is to safeguard the multifragmentary and nondisplaced talus fracture (body and neck) against secondary dislocation in a navigated and minimally invasive manner using screw osteosynthesis.

**Indications:**

Due to the young age of the patient in the presented case and the risk of a possible secondary dislocation, the decision was made in favor of surgical treatment.

**Contraindications:**

Soft tissue swelling, wound infections and allergies to the osteosynthesis material.

**Surgical technique:**

The video is available online (in English) and shows the individual surgical steps in detail.

Preoperative computed tomography (CT) imaging and screw planning. Attachment of the reference array. 1) Cone beam CT (CBCT) scan, image fusion and fusion control. Planning of the minimally invasive skin incisions. Skin incision, navigated drilling and insertion of the K‑wires. 2) CBCT scan and position check of the K‑wires, fine adjustment if necessary. Insertion of the screws. 3) CBCT scan with subsequent position check of the screws, retightening of the screws if necessary.

Performed in the Robotic Suite (Brainlab, Munich, Germany) using the following elements: navigation unit curve navigation system, movable robotic 3D CBCT, “Loop-X” and wall monitor “BUZZ”.

**Follow-up:**

Postoperative X‑ray and CT to control the position of the implants. Partial weight-bearing of the foot with 10 kg sole contact for 6 weeks. Physiotherapy with active and passive joint mobilization. Thrombosis prophylaxis with enoxaparin sodium.

Optional implant removal after approximately 1 year.

**Evidence:**

Navigated operations are routine, so far mainly in the area of the spine. This article shows that navigated extremity surgery can be successfully performed in hybrid operating theaters.

## Hintergrund

Die operative Versorgung von Talusfrakturen kann sich als relativ kompliziert darstellen. Dies liegt vor allem an begrenzten Möglichkeiten zur Bildgebung. Mit dem C‑Bogen können der Fuß und seine komplexe Anatomie intraoperativ oftmals für die Schraubenosteosynthese nicht ausreichend dargestellt werden. Die operative Versorgung von Verletzungen am Fuß kann sich dadurch sehr langwierig gestalten, mit viel Strahlenbelastung für das Operationsteam einhergehen und mit einem nichtzufriedenstellenden Ergebnis enden.

Bereits in anderen Bereichen der Unfallchirurgie kommen Navigationsmethoden zum Einsatz, v. a. im Bereich der Wirbelsäule [10, 12, 14, 18, 19]. Auch bei anderen Indikationen bietet die Navigation gute Möglichkeiten, eine adäquate operative Versorgung durchzuführen [1, 8, 16, 20]. Wegen den bereits beschriebenen möglichen Problemen bei der Versorgung von Fußverletzungen berichten wir über die Möglichkeit einer navigierten und minimalinvasiven Schraubenosteosynthese am Talus. Diese wurde mittels intraoperativer Cone-Beam-CT(CBCT)-Bildgebung und darauf basierender Navigation durchgeführt.

## Definitionen und Klassifikationen

Wie bereits durch vorhergehende Literaturquellen angemerkt wurde, stellt die Talusfraktur im Gesamtvergleich eine relativ seltene Verletzung dar [4, 13, 15]. Talusfrakturen werden nach Marti [9] in 4 Typen eingeteilt. Dabei werden die Frakturen anhand der Lokalisation sowie anhand des Nekroserisikos eingeteilt. Das Nekroserisiko steigt vom Typ I bis zum Typ IV nach Marti an. Eine operative Therapie sollte aufgrund der Nekrosegefahr bei Typ-III- und bei Typ-IV-Frakturen nach Marti erfolgen [2, 9].

Frakturen des Talushalses werden nach der Hawkins-Klassifikation [6] eingeteilt in 3 Typen, welche später durch Canale und Kelly [3] um einen 4. Typ ergänzt wurden. Die Hawkins-Klassifikation [6] unterscheidet die Frakturen des Halses auf Basis deren Verlaufs sowie auf Basis deren Dislokationsgrades [7]. Die Unterscheidung besitzt auch hier eine Wichtigkeit wegen des angenommenen Risikos einer Nekrose des Talushalses, durch die mögliche Beeinträchtigung der Durchblutung [7, 17]. Generell steigt das Risiko einer möglichen Nekrose, wie bei der Klassifikation nach Marti [9], von Typ I bis Typ IV nach Hawkins an. Damit steigt auch die Notwendigkeit einer operativen Versorgung der Fraktur, wobei Typ-III- und Typ-IV-Frakturen auch hier operativ behandelt werden sollten [2, 6, 7, 17].

Generell sollte bei instabilen und/oder dislozierten Frakturen eine operative Therapie durchgeführt werden. Hierbei stellt bisher die offene Reposition und interne Fixierung („open reduction and internal fixation“, ORIF) die Methode der Wahl dar [2].

## Operationsindikation

In dem für diesen Videobeitrag ausgewählten Fall stellt sich die Operationsindikation durch die mehrfragmentäre Fraktur und das junge Alter des Patienten. Die Operation stellt eine Absicherung der Fraktur gegenüber einer möglichen sekundären Dislokation dar. Die Gefahr von Dislokationen wurde aufgrund der vielen Fragmente sowie der körperlichen Einschränkungen durch die multiplen erworbenen Verletzungen bei einer konservativen Therapie als durchaus realistisch angesehen.

## Fallbeschreibung

Nach einem Verkehrsunfall mit einer Aufprallgeschwindigkeit von ca. 50 km/h wurde ein 20 Jahre alter Patient mit multiplen Verletzungen in den Schockraum unseres Klinikums eingeliefert, darunter eine Talusfraktur rechts sowie eine Talusfraktur links. Der Injury Severity Score (ISS) dieses Patienten lag bei 22. Durch und nach der Schockraumversorgung wurde der Patient stabilisiert und auf die Intensivstation verlegt. Nach der Stabilisierung der Vitalfunktionen wurden weitere diagnostische Schritte für die bereits im Ganzkörper-CT festgestellten Läsionsbereiche eingeleitet. So wurden am rechten Fuß eine konventionelle Röntgendiagnostik sowie eine CT-Bildgebung durchgeführt. Aus der Bildgebung ergab sich die Bestätigung einer Läsion im Bereich des rechten Talus, mit einer mehrfragmentären und undislozierten Talusfraktur (Corpus und Hals), welche eine Typ-II-Fraktur nach Marti bzw. im Bereich des Talushalses eine Typ-I-Fraktur nach Hawkins darstellt. Die Operation wurde nach der Stabilisierung des Patienten und der Normalisierung der Weichteilverhältnisse eine Woche nach dem Unfalltag durchgeführt. Für die peripheren Absprengungen am kontralateralen linken Talus wurde hingegen eine konservative Therapie eingeleitet.

## Operationstechnik

Navigierte Operationen, wie in diesem Beitrag beschriebenen, werden in unserem Klinikum in der „Robotic Suite“, einem Hybrid-OP, durchgeführt. Diese besteht aus der Navigationseinheit „Curve Navigation System“, aus einem fahrbaren robotischen 3D-Computertomographen (CBCT), einem „Loop-X“, einem Wandmonitor „BUZZ“, Mixed-Reality-Brillen und einem robotischen Arm „Cirq Arm System“ (Fa. Brainlab, München, Deutschland). Für den nachfolgend beschriebenen Eingriff wurden das Cone-Beam-CT, die Navigationseinheit und der Wandmonitor verwendet. Die verwendeten Produkte sind für die Unfall- und Wirbelsäulenchirurgie zugelassen. Für die Osteosynthese wurden Schrauben der Fa. Königsee verwendet (Fa. Königsee Implantate, Allendorf, Deutschland).

Die Vorbereitung der Operation beginnt mit der Planung auf Basis des präoperativen CT-Bildes (0:19 min). In diesem ausgewählten Fall wurden 3 Schrauben geplant (4 × 55 mm, 4 × 47 mm und 4 × 20 mm).

Die Operation wird in Rückenlage auf einem Carbontisch durchgeführt. Das fahrbare Cone-Beam-CT ist dabei am Fußende des Patienten platziert.

Als Erstes wird die Patientenreferenzeinheit mittels zwei kleiner Stäbe am Kalkaneus des Patienten befestigt und ausgerichtet (0:37 min). Hierbei ist es wichtig zu planen, dass die Referenzeinheit und die Kamera über die ganze Dauer der Operation Sichtkontakt haben.

**Abb. 1:**
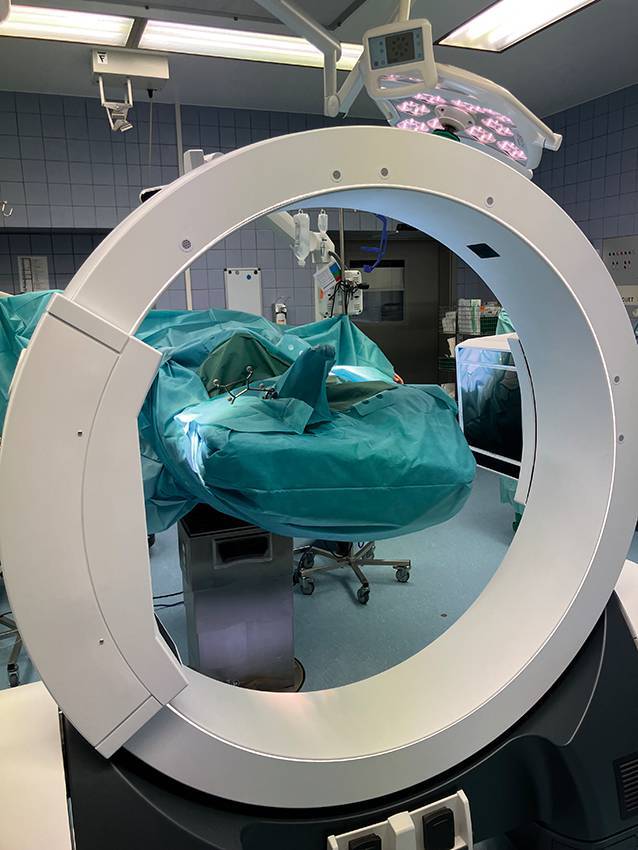
Das Cone-Beam-CT (CBCT) in Scan-Position

Anschließend werden die Vorbereitungen zur ersten CBCT-Bildgebung getroffen. Nach dem Abdecken des OP Bereichs (1:30 min) folgt die Feldaufnahme (1:39 min). In dieser wird kontrolliert, ob das CBCT richtig positioniert ist, um während des späteren Scannens den gewünschten Bereich vollständig abbilden zu können (Abb. 1). Als nächster Schritt folgt die Kollisionsprüfung (1:41 min). Ist diese abgeschlossen, kann nun der eigentliche CBCT-Scan durchgeführt werden (1:49 min). Nun folgt die Fusion des präoperativen CT mit der intraoperativen Bildgebung (1:53 min). Die Kontrolle der Fusion erfolgt visuell auf dem Bildschirm und auch manuell mittels des Pointers (2:20 min). Wichtig ist es, die Position des Operationstisches sowie die Position des CBCT nach dem ersten Scan für die Folgescans zu speichern.

Nach erfolgreicher Fusion können die minimalinvasiven Hautschnitte geplant werden (2:34 min).

**Abb. 2:**
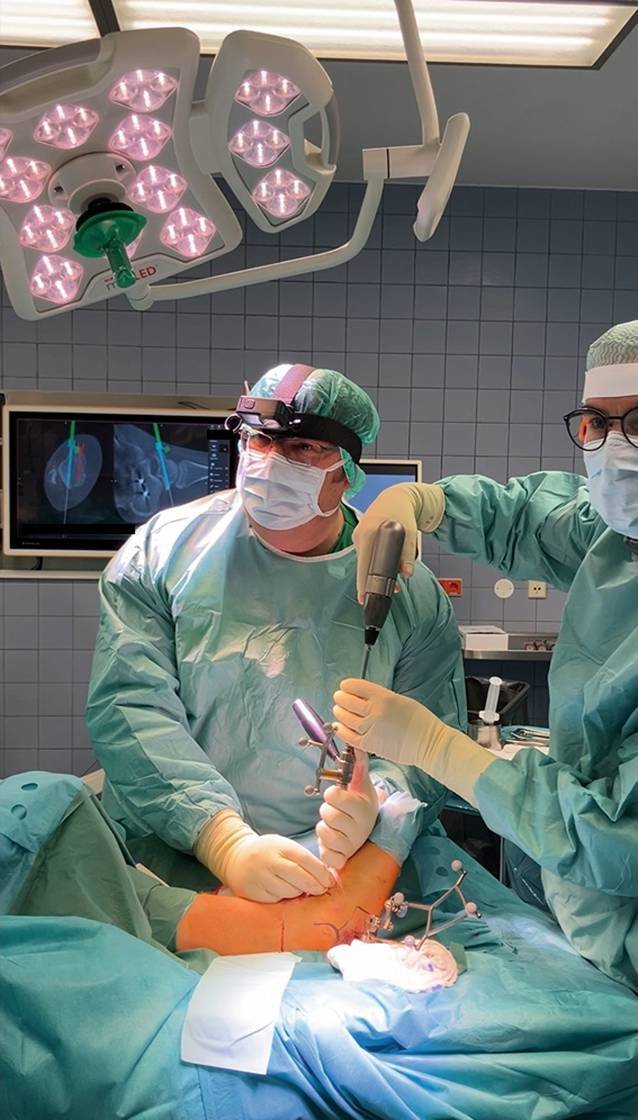
Durchführung der navigierten Bohrung

Nun werden die zu verwendenden Instrumente referenziert (2:58 min) und die Hautschnitte durchgeführt (3:05 min). Für die navigierte Bohrung wird die Bohrhülse mit Blick auf den Bildschirm ausgerichtet und entsprechend dem geplanten Trajekt platziert (3:19 min). Dann folgen die Bohrung (Abb. 2) und das Einführen der K‑Drähte in das Bohrloch.

Nach diesem Schritt wird der zweite CBCT-Scan zur Lagekontrolle der Kirschner(K)-Drähte durchgeführt (4:36 min). Wenn nötig, können die K‑Drähte weiter eingebracht werden, unter Abmessung der Wegstrecke auf dem Bildschirm.

Wenn die Lage der K‑Drähte zufriedenstellend ist, können nun die Schrauben eingebracht werden (5:10 min).

Es folgen der dritte CBCT-Scan und die Lagekontrolle der Schrauben (5:22 min). Auch hier kann die Einbringtiefe der Schrauben noch angepasst werden. Wenn sich die Schrauben in einer zufriedenstellenden Lage befinden, können die K‑Drähte sowie die Referenzeinheit nun entfernt werden.

Es folgen der sterile Wundverschluss und das Anbringen eines sterilen Wundverbandes.

**Abb. 3:**
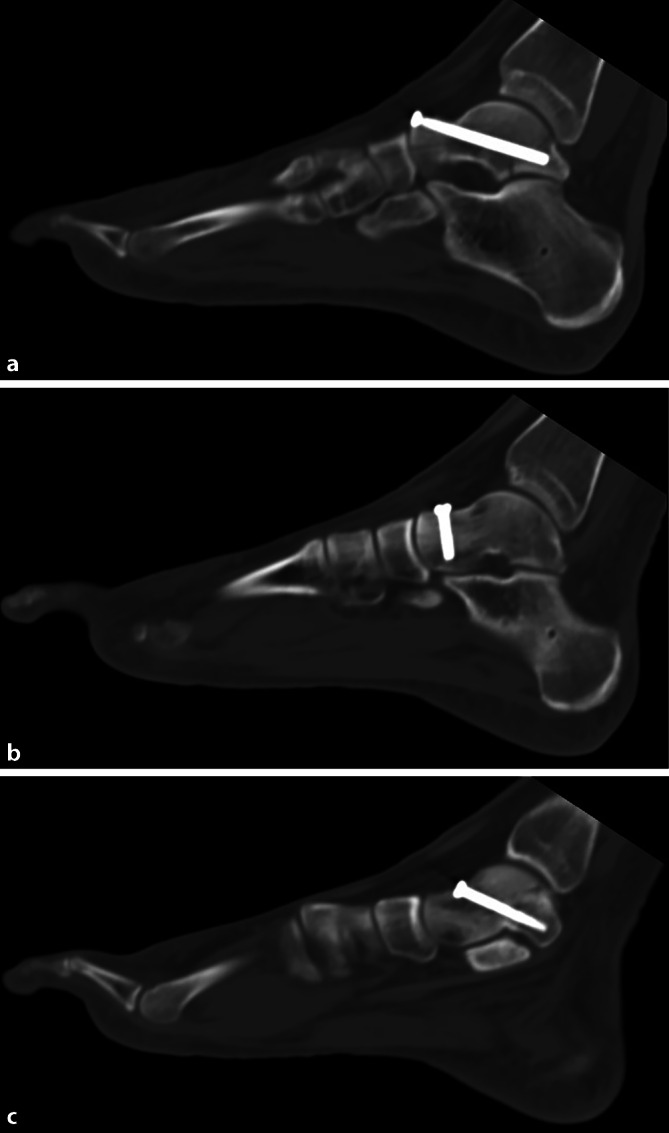
**a**–**c** Postoperative Kontroll-CT (**a** multiplanare Rekonstruktion sagittale Ebene Schraube 1; **b** multiplanare Rekonstruktion sagittale Ebene Schraube 2; **c** multiplanare Rekonstruktion sagittale Ebene Schraube 3)

Postoperativ erfolgte eine Lagekontrolle der Implantate mittels Röntgen und CT (5:33 min), dabei zeigte sich eine sehr gute Implantatlage und somit ein sehr zufriedenstellendes Operationsergebnis (Abb. 3).

## Postoperative Behandlung

Für den hier beschriebenen Fall ergab sich das nachfolgend beschriebene postoperative Vorgehen.

Für den ersten postoperativen Tag wurde eine Röntgenkontrolle (rechter Fuß: oberes Sprunggelenk, Kalkaneus tangential) sowie eine CT-Kontrolle angeordnet (Abb. 3).

Postoperativ empfahlen wir die Kühlung und Hochlagerung des betroffenen Fußes. Es wurde eine Schmerzmedikation mit nichtsteroidalen Antirheumatika (NSAR) nach Bedarf angeordnet.

Für 6 Wochen sollte eine Belastung von 10 kg bei Sohlenkontakt mit Gehilfen erfolgen. Nach diesen 6 Wochen sollte die Belastung schrittweise bis zur Vollbelastung gesteigert werden. Physiotherapeutisch wurde eine aktive und passive Gelenkmobilisation angeordnet. Zur Thromboseprophylaxe wurde Enoxaparin-Natrium (4000 IE/s.c. einmal täglich) bis zur Vollbelastung angeordnet.

Die Metallentfernung ist fakultativ und im Verlauf nach ca. einem Jahr möglich.

## Fehler, Gefahren und Komplikationen

Von den chirurgischen Risiken unterscheiden sich navigierte Techniken nicht grundlegend von den konventionellen Techniken. Die Beherrschung konventioneller Techniken ist essenziell, um die Vorteile von navigierten Techniken optimal nutzen zu können. Die Risiken und Gefahren sind v. a. im Umgang mit der Technik oder bei der Technik selbst zu sehen. Folgende Bereiche gelten oftmals generell für navigierte Operationen und nicht ausschließlich bei navigierten Eingriffen am Talus.

Allen voran können technische Probleme unter der Nutzung moderner Techniken auftreten, verschuldet oder unverschuldet durch das OP-Team. Hier sollte der Chirurg im Vorhinein auf alternative chirurgische Optionen vorbereitet sein. Für diesen Fall ist der Ausdruck von Schraubenplanungen und die Platzierung sichtbar im OP eine gute Sicherheitsmaßnahme. So besteht, egal bei welcher Art von möglichen unerwarteten Problemen, die Möglichkeit, auf die konventionelle Technik umzustellen, ohne die Operation abbrechen zu müssen. Im Falle technischer Probleme ist es außerdem sinnvoll, die Erreichbarkeit des Herstellers im Vorhinein sicherzustellen oder Fachpersonal bei der Operation anwesend zu haben. Die Schulung des OP-Teams ist essenziell vor dem Einsatz solcher Techniken, sodass keine menschlichen Fehler die Operation beeinflussen. Mögliche Szenarien stellen hier die Verschiebung oder Diskonnektion der Patientenreferenzeinheit sowie eine Fehlbedienung (Kollision und Gefährdung des sterilen Feldes) des Cone-Beam-CT dar.

Die potenziell größte Fehlerquelle einer navigierten Operation kann die Fusion von präoperativen Bildern mit der intraoperativen Bildgebung darstellen. Für diesen Schritt sollte ausreichend Zeit eingeplant werden und bei Zweifeln eine erneute Fusion durchgeführt werden, da eine fehlerhafte Fusion sehr große Auswirkungen haben kann. Am Ende der Fusion sollte daher auch in jedem Falle eine Kontrolle mit dem Pointer durchgeführt werden, um das Ergebnis der Fusion nochmals eigenständig auf die Korrektheit zu überprüfen.

Abschließend gilt es hervorzuheben, dass die Schulung und Vorbereitung des Teams die Gefahren und Probleme minimieren. Nur bei ausreichender Kenntnis des Systems sowie bei einem optimalen Arbeitsablauf kann eine navigierte Operation am Talus erfolgreich durchgeführt werden.

## Evidenz der Technik

Die Verwendung von Navigationstechniken ist weit verbreitet; in der Orthopädie und Unfallchirurgie wird die Navigation bisher überwiegend im Bereich der Wirbelsäule verwendet [5, 10–12, 14, 18, 19]. Zunehmend erweitern sich die Anwendungsbereiche auch auf andere Eingriffsgebiete am gesamten Bewegungsapparat [1, 8, 16, 20].

In unserer Klinik wurden bisher über 260 navigierte Operationen in diesem hier verwendeten Hybrid-OP durchgeführt. Die Eingriffsbereiche lagen hierbei an der Wirbelsäule, am Becken und an den Extremitäten. Das hier verwendete Videomaterial entstand im Setting einer klinischen Routineoperation.

Dieser Beitrag soll zeigen, dass navigierte Operationen an den Extremitäten in einem 3D-Navigation-Hybrid-OP erfolgreich und mit sehr guten Operationsergebnissen durchgeführt werden können.

## Fazit für die Praxis

- Talusfrakturen kommen epidemiologisch eher selten vor.
- Die Gefahr einer Nekrose ist je nach Frakturtyp gegeben, und Therapieentscheidungen sollten daher zügig getroffen werden.
- Eine operative Versorgung des Talus ist v. a. im Hinblick auf die intraoperative Bildgebung anspruchsvoll.
- Eine navigierte Operation am Fuß bedarf einer ausführlichen Vorbereitung und eines gut strukturierten Arbeitsablaufes.
- Ein Rückfallplan ist in jedem Fall sinnvoll, und konventionelle Operationsmethoden sollten vom Operateur beherrscht werden.
- Nach der navigierten Bohrung und des Einbringens der Kirschner-Drähte folgt eine Lagekontrolle, bevor die Schrauben eingebracht werden.
- Navigierte Eingriffe am Fuß bringen den Vorteil mit sich, dass diese minimalinvasiv und mit einer sehr hohen Präzision durchgeführt werden können.
- Navigierte Eingriffe an den Extremitäten können erfolgreich und mit sehr guten Ergebnissen in einem 3D-Navigation-Hybrid-OP durchgeführt werden.

## Supplementary Information

Operationstechnik der navigierten und minimalinvasiven Schraubenosteosynthese einer Talusfraktur; angenommen: 18-11-2024
